# Current Research Focus and Trends of Remimazolam: A Bibliometric Analysis of the 100 Most Cited Articles

**DOI:** 10.2174/011570159X370775250704060228

**Published:** 2025-07-24

**Authors:** Yunying Chen, Junting Wu, Huangyi Chen, Chenxing Lei, Dezhao Liu, Ying Wang

**Affiliations:** 1 Department of Anesthesiology, The Fifth Affiliated Hospital, Sun Yat-sen University, Zhuhai, 519000, China

**Keywords:** Bibliometric analysis, bibliometrics, remimazolam, most-cited articles, Web of Science, VOSviewer, CiteSpace

## Abstract

**Introduction:**

Remimazolam is a novel benzodiazepine derivative with advantages such as prompt onset, short duration of action, fast recovery, and non-organ dependence. Numerous studies have been conducted on remimazolam. However, bibliometric analysis on high-quality and highly cited articles related to remimazolam is lacking. The objective of this article is to evaluate the current research status and prevailing trends regarding the most frequently cited articles on remimazolam, utilizing bibliometrics.

**Methods:**

Studies related to remimazolam were searched in the Web of Science core database. The search period ranged from the inception of the database to April 2025, and 100 highly cited research articles were selected. The researchers gathered and analyzed pertinent data from the studies and subsequently conducted visual analysis utilizing VOSviewer and CiteSpace.

**Results:**

The total number of citations for the top 100 highly cited studies was 6683, published between 2010 and 2024. China, the United States, and the United Kingdom contributed the majority of these studies. These studies were published in 47 different journals. The journal with the highest number of publications was the *Journal of Anesthesia*. The institution with the highest publication volume was PAION DEUTSCHLAND GMBH in Germany, and the author with the highest contribution was Schippers F. The pharmacokinetics, pharmacodynamics, safety, and efficacy of remimazolam were the main research directions and focuses in the field.

**Discussion:**

Our analysis of the top 100 cited remimazolam papers reveals a rapidly advancing field. The surge in high-quality clinical studies confirms remimazolam's practical edge over older agents, such as propofol, particularly in offering better blood pressure stability for older patients and fewer breathing problems during procedures like endoscopy. While these advantages position it as a strong contender, important questions linger about its use in people with severe liver or kidney issues due to how it is broken down, and its effects on delirium remain unclear. Broadening research globally and focusing on these specific patient groups, as well as long-term safety, will be key to realizing remimazolam's full clinical potential.

**Conclusion:**

This study analyzed the 100 most frequently referenced articles on remimazolam, providing valuable insights into the characteristics and focal areas of research related to this topic.

## 1 INTRODUCTION

Remimazolam is an innovative benzodiazepine derivative that exerts its pharmacological effects by modulating γ-aminobutyric acid receptors. It possesses not only the benefits of rapid onset, brief duration of action, and independence from organ reliance, but also offers the advantage of swift recovery, facilitated by its breakdown by tissue esterases, thereby bypassing the need for liver enzymes [1, 2]. Additionally, its effects can be promptly reversed with the concurrent administration of the benzodiazepine receptor antagonist known as flumazenil [3, 4]. Compared with midazolam and propofol, remimazolam demonstrates superior controllability [5-8]. Specifically, its hemodynamic stability demonstrates significant improvement over propofol, with a 30-50% reduction in the incidence of hypotension during the induction period [9]. The agent exhibits a more favorable respiratory depression profile and lower incidence of adverse reactions, including the absence of injection pain associated with propofol administration [10, 11].

Furthermore, remimazolam maintains anterograde amnesia effects comparable to those of midazolam [12]. The drug has been approved since 2020. It was first approved for general anesthesia in Japan in January 2020, for induction and maintenance of sedation in the United States in October 2020, and for sedation during colonoscopy and induction of general anesthesia in China in July 2020 [13, 14].

Recent clinical studies have highlighted that remimazolam offers a superior safety profile compared to etomidate in cardiac surgery patients, particularly in maintaining hemodynamic stability and minimizing the risk of adrenal suppression [15]. In pediatric settings, emerging evidence highlights its clinical utility across various high-risk populations, including effective anesthesia management in children with Duchenne muscular dystrophy [16], safe use in patients susceptible to malignant hyperthermia [17, 18], and efficacy in preventing emergence delirium during tonsillectomy procedures [19]. Nonetheless, the current research is limited by small sample sizes in most trials. The restricted generalizability of these findings, along with unresolved questions regarding long-term safety, underscores the need for large-scale clinical trials to establish comprehensive, evidence-based guidelines [20].

In current research, evaluating the quality and quantity of literature is becoming increasingly important, and bibliometric analysis is the most important method [21]. Bibliometrics integrates large-scale literature data and quantitatively analyzes published papers and their attributes to identify bibliometric characteristics of publications in a given field, thereby objectively revealing research trends and knowledge structures [22, 23]. Additionally, statistical data is used to describe or show the relationships among the published work [24, 25]. Selecting the study with the highest citation count from the searched literature list is one of the most common methods in bibliometric analysis [26-28]. The advantages of analyzing highly cited literature have been fully verified in the fields of anesthesiology and pharmacology. For instance, Chen *et al*. successfully mapped out the research hotspot map of postoperative analgesia and perioperative complications by analyzing high-impact anesthesiology papers in the past decade. And identify the core academic forces and cross-institutional cooperation models of countries such as the United States and the United Kingdom [29].

Currently, a significant number of studies on remimazolam have been published, encompassing bibliometric analyses conducted within a specified time range in the field of remimazolam [30]. Overall, there is an upward trend in the amount of literature published. However, a dearth of bibliometric analysis pertaining to remimazolam can be observed in the existing literature, particularly concerning articles of superior quality and significant citation rates. Analyzing highly cited studies can provide insights into the progress and important achievements in the field [31], so the main objective of this study is to qualitatively and quantitatively analyze 100 highly cited articles in the field of remimazolam, aiming to facilitate researchers in comprehending the current research focus and significant research outcomes in this field, and to furnish references for future in-depth investigations in this area.

## 2 MATERIALS AND METHODS

### 2.1 Search Strategy

In the Web of Science database, selecting the “Topic” search category will retrieve relevant literature by searching across the titles, abstracts, and indexing of all publications in the database. Relevant literature was searched from Web of Science core databases on April 2025, using the search term combination “remimazolam (Topic)” OR “CNS7056 (Topic)” OR “remimazolam besylate (Topic)” OR “remimazolam tosilate (Topic)” as a search strategy to retrieve studies in the field of remimazolam. The retrieved literature was arranged in descending order of citation frequency.

### 2.2 Literature Inclusion

To minimize bias, two researchers independently searched the Web of Science core databases to derive a list of the most frequently cited articles, and after comparing the lists for discrepancies, each identified literature was evaluated by reviewing the article titles, abstracts and full text if necessary to ensure that the content of the article was relevant to remimazolam. When two or more articles have an equal number of citations, they are ranked equally. If there are more than 100 articles, the ranking is adjusted based on the average annual citation frequency per article. Ultimately, the top 100 most cited relevant articles were selected, and key information, including authors, affiliations, titles, publication years, citation frequencies, journal names, impact factors (IF), and countries or regions, was extracted.

### 2.3 Statistical Analysis and Visualization Analysis

After collecting literature data from the Web of Science Core Collection, we employed multidimensional analysis to uncover the developmental trends within various disciplines. The associations between predictors and citation counts were assessed using univariate analysis followed by multivariate negative binomial regression, with variables screened at *p* < 0.1 Statistical charts, such as bar charts and trend curves, were generated using GraphPad Prism 8 to standardize the bibliometric characteristics. Additionally, the dynamic evolution of prominent topics was analyzed using VOS viewer 1.6.20, in conjunction with time-slicing technology. Furthermore, CiteSpace 6.3.2 was utilized for Burst Detection to identify the intensity and temporal evolution of emerging topics.

## 3 RESULTS

### 3.1 Article Type, Language, Year of Publication, and Citation Trends

**Fig. (2):**
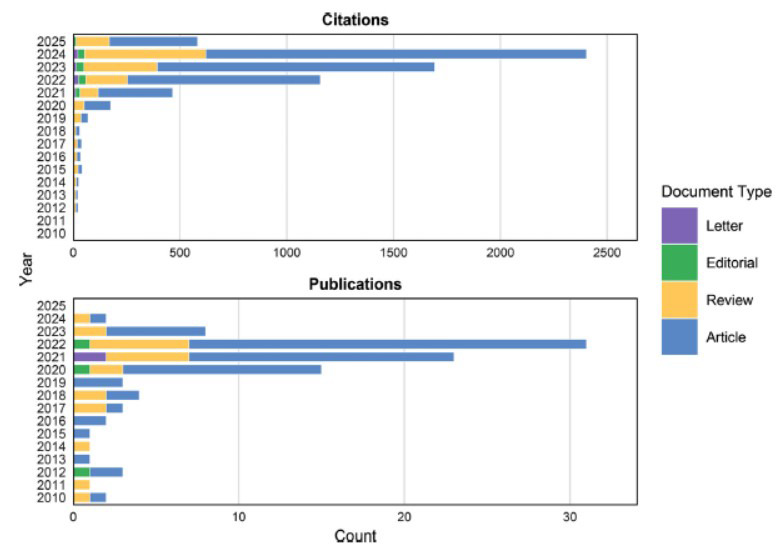
Annual publications and citations of the top 100 articles of different document types. This figure illustrates the annual trends in academic publications and citations from 2010 to 2025. Publication counts exhibit continuous growth peaking in 2024, while citations surge markedly in later years, reaching maximum values by 2024. The bottom section presents the document type distribution, revealing the proportions of different research formats.

A total of 918 articles were retrieved from the Web of Science core database. Fig. (**1**) illustrates the trend of literature publication on remimazolam in the WOS database from 2010 to 2025. Overall, there is an upward trend in the amount of literature published, which serves as evidence of a significant research inclination towards remimazolam. From these 918 articles, we extracted the top 100 articles with the highest citations for research. The articles are categorized into four main types: original articles (n = 72), reviews (n = 23), editorial materials (n = 3), and letters (n = 2). All of the top 100 most-cited articles were published in English. The articles were published between 2010 and 2024, with the highest number of publications in 2022 (n=31), followed by 2021 (n=23) and 2020 (n=15) (Fig. **2**). In terms of citation frequency, there has been a steady increase in citations from 2013 to 2024 (Fig. **2**). The articles have been cited a total of 6690 times, excluding self-citations which account for 5897 citations. The average number of citations per article is 66.9, while the average h-index is 49. The year with the highest citation frequency is 2024, with a total of 2403 citations.

### 3.2 Geographic Distribution

**Fig. (3):**
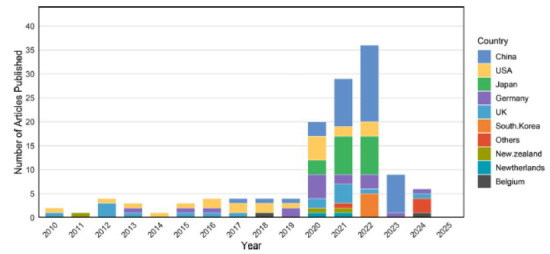
Geographic distribution of the top 100 most cited articles. This visualization displays the distribution of academic article publications by country (including China, USA, Japan, Germany, *etc*.) from 2010 to 2025. The horizontal axis denotes years, and the vertical axis represents publication counts, with countries differentiated by color. China and the United States maintain publication leadership, reaching peak outputs in 2022, while other nations, such as South Korea and Germany, demonstrate stable growth trajectories.

Geographically, the 100 most cited articles originated from 15 countries or regions (Fig. **3**). Mainland China was the most prolific region, with 38 articles, followed by the United States (n=22), Japan (n=19), Germany (n=17), and the United Kingdom (n=16).

### 3.3 Contributing Institutions

Fig. (**4**) displays the institutions that made a significant contribution to the 100 most cited articles about remimazolam. Only those institutions that had published four or more articles are included in it. Among these 13 institutions, 8 are from China, while the remaining are from Germany, the United Kingdom, Netherlands, Japan and the United States. PAION DEUTSCHLAND GMBH from Germany had the highest number of publications (n=9), followed by PAION UK LTD (n=8) from the United Kingdom.

### 3.4 Authors Analysis

The quantity of articles authored by an individual serves as an indicator, to a certain degree, of the author's contribution and engagement in this field. There are 506 authors in the top 100 most frequently cited articles on remimazolam, and authors with four or more publications are listed in Table **1**. Schippers F. from Germany emerges as the most prolific author, with his name being featured in 9 out of the top 100 articles. The total citation frequency for these articles is 687, excluding 684 times of self-citations. The average citation frequency per article is 85.88, with an h-index of 8. Authors ranked second through sixth were Borkett KM. (n=5), Sneyd Jr. (n=5), Ihmsen H. (n=4), Jiang J. (n=4), and Stöhr T. (n=4).

### 3.5 Journal Analysis

The articles were published in 47 different journals, listed in descending order. The IF and JCR areas of articles are specifically highlighted in Table **2**. According to the JCR categories, 18 journals were classified in Q1, 20 journals in Q2, and 9 journals in Q3. There are 10 journals with a citation frequency of more than 2. The most frequently cited journals were the *Journal of Anesthesia* (n=13), followed by *Drug Design, Development, and Therapy* (n=9) and *BMC Anesthesiology* (n=8). According to the journal citation report published in 2024, *Pharmacological Reviews* had the highest IF of 19. followed by *Drugs* (IF=13) and *Chest* (IF=9.5). In addition, 10 journals demonstrated IFs exceeding 5.

### 3.6 Analysis of Citation-related Factors

Multivariate negative binomial regression showed significant effects of document type and categories (Table **3**). Reviews (*vs*. Articles: (Incidence Rate Ratio, IRR) = 2.15, *p* < 0.001) and Pharmacology & Pharmacy (*vs*. Anesthesiology: IRR = 1.53, *p* < 0.001) increased citations, while Letters (*vs*. Articles: IRR = 0.23, *p* = 0.001) decreased citations. Years since publication and country had no significant effects.

### 3.7 Analysis of the 10 Most Cited Articles

Details of the 10 most cited articles can be found in Table **4**. The articles encompassed a period from 2012 to 2020 and comprised a total of nine primary research papers and one literature review, all written in English. The collection of nine primary research papers primarily examined the safety and effectiveness of remimazolam, with two articles dedicated to each phase of clinical trials (phases I, IIb, and III). The sole review article evaluates the literature on the clinical use of remimazolam, compares it with existing sedatives, and describes its potential role in sedation.

### 3.8 Keyword Visualization and Analysis

**Fig. (5):**
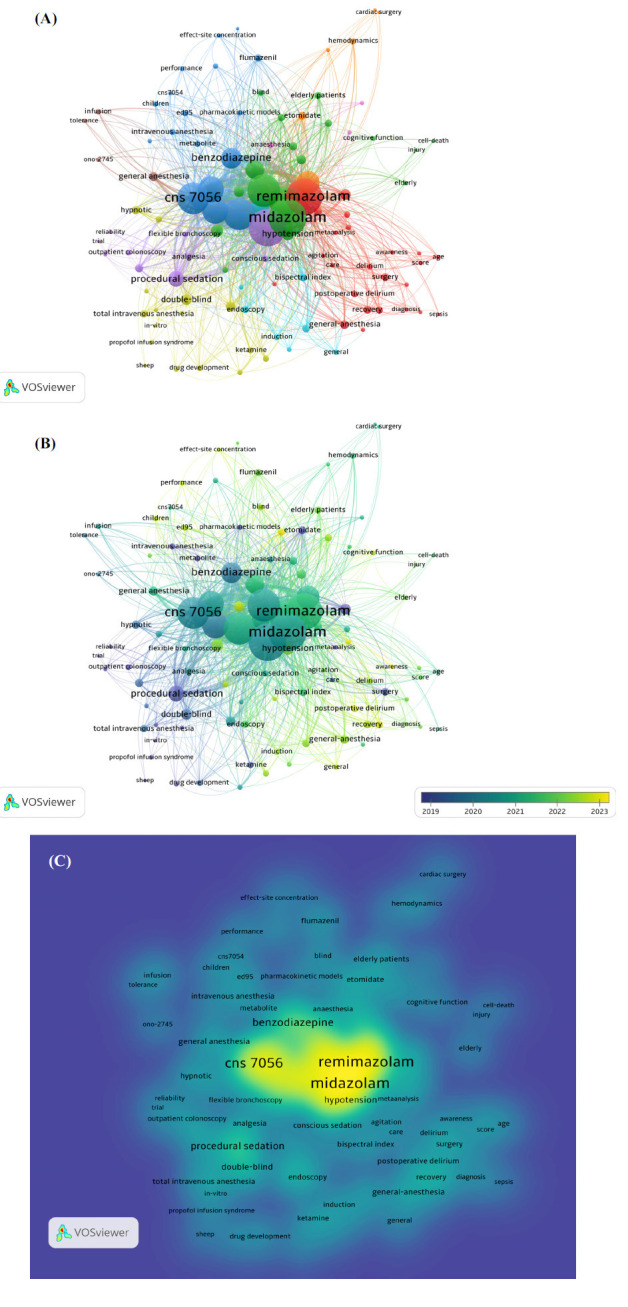
Co-occurrence of the keywords of the top 100 most cited articles. (**A**) presents a co-occurrence network of keywords, where different colors represent distinct clusters. Keywords like “remimazolam” and “midazolam” are centrally located, highlighting their importance in the research. (**B**) is similar to Figure A but shows changes over time, with colors transitioning from blue to yellow to indicate this progression. (**C**) shows a density map of keywords, where darker colors indicate higher frequency in the literature. “Remimazolam” and “midazolam” remain core keywords, reflecting significant research interest in these areas.

The analysis of keywords present in the authors' publications and those in WOS (KeyWords Plus) publications was conducted using the VOSviewer software. This study manually identified keywords with semantic relevance and constructed their co-occurrence network using a network visualization graph (Fig. **5A**). The size of the circles represents the frequency of occurrence of each keyword. Larger circles indicate a higher frequency of keyword usage. For example, “remimazolam” is the most frequently used keyword, followed by “midazolam”. The most commonly used keywords are “propofol”, “sedation”, and “safety”. In addition, all these selected keywords can be roughly categorized into four clusters, denoted by the colors red, yellow, green, and blue. Different colors are used to indicate the average publication year of the identified keywords, wherein a greater degree of yellow hue is assigned to keywords published subsequent to 2022 (Fig. **5B**). The density visualization map depicted illustrates the mapping of identified keywords according to their appearance frequency. The yellow-colored keywords have the highest appearance frequency, as determined by the average distribution of appearance frequencies (Fig. **5C**).

**Fig. (6):**
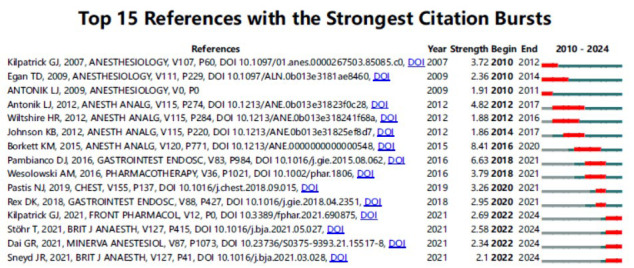
The burst status of the top 15 References with the strongest citation. It presents the 15 most citation-burst literature from 2010-2024 in anesthesia, pharmacology, and medicine (*e.g*., Anesthesiology, Chest). Data reveal persistent disciplinary impacts of seminal works and dynamic evolution of research frontiers.

Furthermore, in addition to examining co-occurrence relationships and clusters, Citespace analysis can also be utilized for detecting bursts in keyword and reference citations, enabling the identification of frequently cited concepts or topics over time. In these analyses, the blue line represents the time interval, while the red segment indicates the period during which the referencing surge occurred. Among the top 100 cited articles, “placebo” ranked first with the highest burst intensity (2.3), followed by “double blind” (1.96) and “procedural sedation” (1.79). The sustained prominence of “elderly patients” (1.39), “gastrointestinal endoscopy” (1.39), and “recovery” (1.21) until 2024 suggests their continued relevance as focal points in contemporary research (Fig. **6**). Regarding the citation burst of references, the most substantial burst value is “A Phase IIa, randomized, double-blind study of remimazolam (CNS 7056) *versus* midazolam for sedation in upper gastrointestinal endoscopy”, which also ranks as the eighth most cited article in the field. The reason Additionally, the authors conducted a comparative analysis between remimazolam and midazolam, further supporting the safety and efficacy of remimazolam for short-term procedures, such as upper endoscopy. It is noteworthy that while the frequency of citations has decreased for some references, four specific references have maintained a high level of impact, suggesting sustained interest in these research topics.

**Fig. (7):**
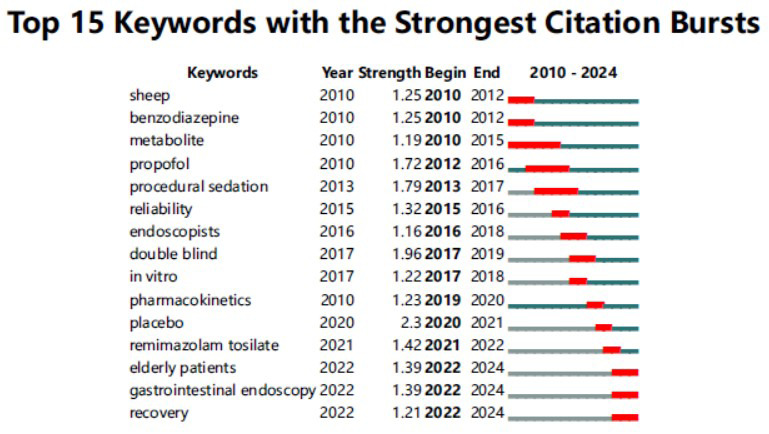
The burst status of the top 15 Keywords with the strongest citation. Early terms, such as “benzodiazepine” (2010-2012), reflect foundational pharmacological research, while later hotspots, including “remimazolam tosilate” (2021-2022) and “elderly patients” (2022-2024), emphasize novel therapeutics and specific patient populations. The prominence of “placebo” (burst strength 2.3) in 2020 highlights the importance of clinical trial design considerations. Data maps the paradigm shift from theoretical exploration to clinical implementation and the emergence of novel research themes.

These four articles systematically examine the pharmacological properties, clinical effects, and appropriateness of remimazolam for special populations. Two of the articles offer specific dosing recommendations and comparative data, while the other two focus on a comprehensive analysis and discussion of trends related to remimazolam. Together, they equip clinicians with multidimensional support, bridging the gap between foundational research and practical application (Fig. **7**).

## 4 DISCUSSION

### 4.1 Bibliometric Analysis

This bibliometric study focuses on the characteristics of the top 100 most frequently cited research articles on remimazolam in the Web of Science Core Collection database. In terms of publication type, more than 72% of the top 100 most cited articles were original articles, 23% were review articles, 3% were editorial materials, and 2% were letters. This suggests that remimazolam is still in a preliminary stage of investigation, and further research is needed to establish a more comprehensive understanding of the drug in new exploratory studies. From the perspective of publication years, the 100 most cited papers were published between 2010 and 2024. The number of papers published from 2010 to 2017 did not exceed 3, but the number of papers published in 2022 began to exceed 30, reaching its highest point.

Furthermore, the overall publication volume on remimazolam has been increasing year by year, indicating that people's research on remimazolam is gradually deepening and expanding in a more comprehensive and in-depth direction. In terms of publishing countries/regions, the Chinese mainland ranked first with a total of 38 publications, followed by the United States (n=22) and Japan (n=19). In addition, PAION DEUTSCHLAND GMBH in Germany published the largest number of papers (n=9), and the first prolific author (n=9) was Schippers F from this institution. At present, the highly cited articles are still concentrated in individual countries, which also leads the current direction of the field. This situation also reflects the uneven progress of research in different regions. The analysis of the journal's 2023 IF and JCR category will also help researchers understand the significance of remimazolam research and make informed decisions on where to submit their work. Over 80% of the submitted journals are situated within the Q1 and Q2 regions of the JCR category, indicating the scholarly and rational quality of the referenced articles. Two articles were submitted to Drugs, a journal with an impact factor of over 10 in 2023. One reviews remimazolam's pharmacodynamics, pharmacokinetic properties, efficacy, and safety in clinical applications [32]. The other article discusses significant milestones in the development of remimazolam leading to its initial approval for general anesthesia induction and maintenance, as well as its forthcoming approval for general anesthesia and surgical sedation [13].

According to the data collected by VOS viewer, based on citation frequency and total link strength, excluding remimazolam itself, the keyword with the highest occurrence frequency and total link strength is midazolam, followed by propofol. In endoscopic surgery, midazolam or propofol are both commonly used as effective intravenous sedatives [33-36]. Midazolam is favored for its rapid onset of action, short duration of effect, low risk of thrombophlebitis, and high amnestic properties [37, 38]. On the other hand, propofol is preferred due to its rapid onset of action and short half-life [39, 40]. However, midazolam has a tendency for significant cumulative effects and relatively longer recovery time [41, 42], while propofol is associated with side effects such as injection pain, hypotension, and respiratory depression [43, 44]. Researchers expected to find more effective and safer drugs, so many articles compare midazolam/propofol with remimazolam in colonoscopy, bronchoscopy, or general anesthesia induction and maintenance to highlight the excellent characteristics of remimazolam, such as rapid onset, rapid metabolism, and non-accumulation [45-49]. From the perspective of time, initial research focused on remimazolam's pharmacokinetics, pharmacodynamics, and safe dose, while later research concentrated on its potential side effects or its effects in specific procedures, such as gastrointestinal endoscopy, indicating a shift in research focus from remimazolam's nature to its characteristics.

### 4.2 Current Research Status and Future Trends

When introducing a new drug, researchers prioritize assessing both its efficacy and safety. At this stage, the hemodynamic effects of remimazolam, which is used for induction and maintenance in general anesthesia, have become the primary focus of research. Several studies have shown that, compared to younger patients, the efficacy of anesthetic drugs in elderly patients is significantly enhanced, resulting in a greater inhibitory effect on respiratory and circulatory functions [50, 51]. In multiple controlled trials, Remimazolam has demonstrated enhanced tolerability in elderly patients [52], with a wide range of effective sedation doses [53] and a reduced incidence of dose-dependent hypotension [54-56]. Furthermore, Remimazolam is more effective in maintaining circulatory stability during surgical procedures compared to propofol [57]. This suggests that Remimazolam may offer advantages for elderly patients, including shorter recovery and extubation times. Not only that, remimazolam's stable hemodynamic characteristics and wide dose range make it a potentially advantageous option for patients with general liver and kidney dysfunction or other severe disease [58]. Randomized controlled studies have shown that remimazolam is associated with a reduced risk of perioperative respiratory depression or hypotension in comparison to propofol [59-61]. Remimazolam not only provides satisfactory anesthesia during surgery but also facilitates quicker recovery when used in combination with flumazenil. However, when talking about patients with severe hepatic and renal insufficiency, clinicians should exercise increased caution with remimazolam due to its metabolism by the non-specific tissue esterase carboxylesterase 1 (CES1). Inhibition of CES1 by substances such as flavonoids, fatty acids, and alcohol can lead to a 300-fold reduction in the affinity of remimazolam metabolites for GABA receptors, thereby prolonging its half-life [3, 62-64]. Singal and Uchida *et al.* reported cases of delayed recovery in patients with severe liver damage [65]. At the same time, a study by Thomas Stöhr *et al*. revealed a threefold increase in metabolite concentration in the urine of patients with end-stage renal disease four hours after receiving a high-dose remimazolam injection compared to healthy individuals [66]. In patients with severe hepatic and renal insufficiency, pharmacodynamic evidence for remimazolam remains limited.

Additionally, the effect of remimazolam on postoperative delirium has become a topic of considerable interest. Researchers are employing various mechanistic models and methodologies to examine how remimazolam may mitigate cognitive dysfunction. Mao *et al.* [67] found that appropriate doses of remimazolam can mitigate histological injury, neuronal apoptosis, microglial activation, and secondary inflammatory responses, thereby improving cognitive dysfunction following cryogenic arrest through the HMGB1-TLR4-NF-κB signaling pathway. The study conducted by Zhan Zhou *et al*. [68] and Leguang Zhou *et al*. [69] corroborated the ability of remimazolam to enhance cognitive function impaired by lipopolysaccharides by activating α7nAChR-mediated Nrf2/HO-1 signaling and regulating transmembrane carrier proteins to inhibit microglial activation, thus recognizing the protective effect of remimazolam on neuronal cells. In clinical studies, Jin-Jin Yang *et al*. [70] demonstrated that remimazolam exhibits a favorable safety profile in elderly patients undergoing orthopedic surgery. However, there was no significant difference in the incidence of postoperative delirium between propofol and the other group. Furthermore, Cai *et al*. [71] reported that among children aged 1 to 6 years, remimazolam administered either as a continuous infusion or as a single dose at the end of surgery significantly reduced the incidence of postoperative delirium compared to placebo during laparoscopic hernia repair surgery. However, there remains no consensus regarding the effect of remimazolam on postoperative delirium, indicating a need for further research in this area.

Remimazolam is increasingly preferred over conventional sedatives in endoscopic procedures owing to its improved safety profile [6, 45]. Evidence demonstrates reduced rates of injection pain, hypotension, and respiratory depression, solidifying its role in modern sedation protocols [72, 73]. Although short-term studies have confirmed their safety and tolerability [74, 75], further investigation is warranted to assess potential variations in safety and efficacy across diverse patient populations based on age, ethnicity, gender, obesity status, body weight, ASA grades, and the occurrence of adverse effects in different types of surgery. Potential adverse reactions and the risks of dependence associated with remimazolam warrant careful consideration. Future research could explore various administration methods and investigate the possibility of combining remimazolam with other medications to mitigate related side effects and promote safer usage.

Furthermore, while remimazolam is currently primarily utilized for intravenous anesthesia and sedation, its potential applications in other clinical areas also require further investigation. In the coming years, researchers from diverse countries will prioritize the expansion and utilization of the drug, the refinement of the field, addressing safety concerns specific to certain populations, optimizing dosage strategies, and personalizing treatment during administration. These areas of focus are of particular interest and ongoing concern for our future research endeavors.

### 4.3 Limitations

This study has several limitations that should be taken into account when interpreting the results. Firstly, the literature search was confined to the Web of Science Core Collection database. Consequently, significant studies published in other databases, such as PubMed and Scopus, may have been overlooked. This limitation could result in an incomplete understanding of the current landscape and trends in remimazolam research. Secondly, the temporal scope of this analysis presents inherent limitations. The evolving nature of scientific inquiry suggests that both the quantity and scholarly impact of remimazolam research will demonstrate chronological variability. As new research emerges, initially under-recognized studies may gain prominence and influence. Therefore, this study can not account for potential shifts in article citations and academic impact following the completion of the search.

## CONCLUSION

In our study, we discovered that the most frequently cited articles on remimazolam were predominantly published between 2010 and 2024. Data from this period indicate a noteworthy shift in research focus. Early investigations focused on the pharmacokinetics, pharmacodynamics, and safe dosage of remimazolam, laying the groundwork for its clinical application. However, recent research has increasingly prioritized evaluating the efficacy of remimazolam in various clinical contexts, its application across diverse medical scenarios, and comparisons with other anesthetics. This trend underscores the sustained global interest in remimazolam, reflecting high expectations for its potential as an anesthetic and sedative. We performed a comprehensive analysis of frequently cited literature to identify key attributes, providing valuable insights into the future research trajectory of remimazolam. These findings not only highlight the current research hotspots and challenges but also suggest directions for future clinical applications and innovative studies.

## AUTHORS’ CONTRIBUTIONS

The authors confirm their contribution to the paper as follows: Study conception and design: WY and LD; data collection: CH and LC; data analysis and interpretation: CY and WJ; methodology: WY and LD; draft manuscript: CY and WJ. All authors reviewed the results and approved the final version of the manuscript.

## Figures and Tables

**Fig. (1):**
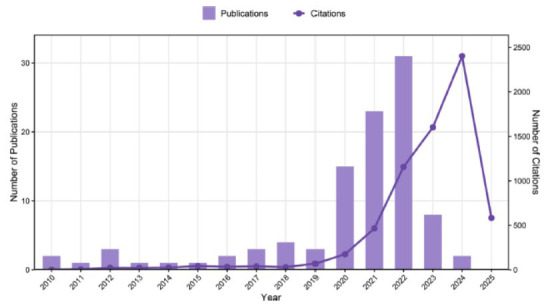
Trends in the number of publications on remimazolam in the WOS database from 2010 to 2025. The publication volume has shown sustained growth since the initial year, while citations demonstrate a significant late-stage escalation, peaking at maximum values.

**Fig. (4):**
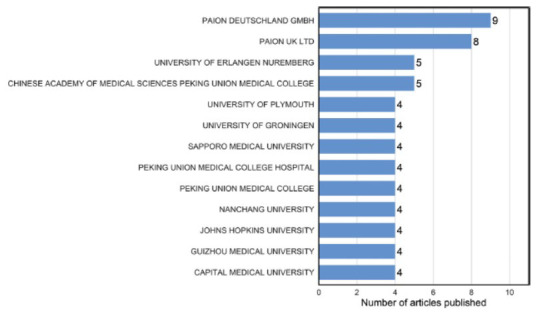
Institutions’ contribution to the 100 most cited articles. This figure identifies the leading global medical research institutions and corporations that contribute to highly cited publications. It visually compares the contributory impact of these organizations through their high-citation academic outputs.

**Table 1:** Authors who published ≥ 4 articles in the top 100 most cited articles.

**Ranking**	**Author**	**Number of Articles**	**Affiliated Organizations**	**Country**
1	Schippers F.	9	Paion Deutschland Gmbh	Germany
2	Borkett KM.	5	Paion UK Ltd	United Kingdom of Great Britain and Northern Ireland
2	Sneyd Jr.	5	University of Plymouth	The United Kingdom
4	Ihmsen H.	4	University of Erlangen-Nuremberg	Germany
4	Jiang J.	4	Chinese Academy of Medical Sciences - Peking Union Medical College	China
4	Stöhr T.	4	Paion Deutschland Gmbh	Germany

**Table 2:** The journals in which the top 100 most cited articles are published.

**Ranking**	**Journal**	**Publication Volume**	**Impact Factor for ** **2023**	**JCR**
1	*Journal of Anesthesia*	13	2.8	Q2
2	*Drug Design, Development, and Therapy*	9	4.7	Q1
3	*British Journal of Anaesthesia*	8	9.1	Q1
4	*Anesthesia and Analgesia*	5	4.6	Q1
4	*Frontiers in Pharmacology*	5	4.4	Q1
6	*BMC Anesthesiology*	4	2.3	Q2
7	*Current Opinion in Anesthesiology*	3	2.3	Q2
7	*Journal of Clinical Anesthesia*	3	5	Q1
7	*Minerva Anestesiologica*	3	2.9	Q1
10	*Anesthesiology*	2	9.1	Q3
10	*British Journal of Clinical Pharmacology*	2	3.1	Q2
10	*Drugs*	2	13	Q1
10	*European Journal of Clinical Pharmacology*	2	2.4	Q3
10	*Frontiers in Medicine*	2	3.1	Q1
10	*Gastrointestinal Endoscopy*	2	6.7	Q1
10	*Journal of Clinical Pharmacy and Therapeutics*	2	2.1	Q3
10	*Journal of Gastroenterology and Hepatology*	2	3.7	Q2
10	*Korean Journal of Anesthesiology*	2	4.2	Q1
19	*American Journal of Translational Research*	1	1.7	Q3
19	*Anesthesia*	1	7.5	Q1
19	*Anesthesia and Pain Medicine*	1	5.1	Q1
19	*Best Practice Research Clinical Anaesthesiology*	1	4.7	Q1
19	*Chest*	1	9.5	Q1
19	*Clinical Interventions in Aging*	1	3.5	Q2
19	*Clinical Therapeutics*	1	3.2	Q2
19	*CNS Drugs*	1	7.4	Q1
19	*Digestive and Liver Disease*	1	4	Q1
19	*Digestive Diseases and Sciences*	1	2.5	Q2
19	*European Journal of Anaesthesiology*	1	4.2	Q1
19	*Frontiers in Aging Neuroscience*	1	4.1	Q2
19	*Idrugs*	1	2.328	Q2
19	*International Immunopharmacology*	1	4.8	Q2
19	*International Journal of General Medicine*	1	2.1	Q2
19	*JA Clinical Reports*	1	0.8	Q3
19	*Journal of Bronchology Interventional Pulmonology*	1	3.3	Q2
19	*Journal of Clinical Pharmacology*	1	2.4	Q3
19	*Journal of Oral Science*	1	1.9	Q2
19	*Journal of Pharmaceutical and Biomedical Analysis*	1	3.1	Q2
19	*Journal of Pharmacy and Pharmacology*	1	2.8	Q2
19	*Medicine*	1	1.3	Q2
19	*PeerJ*	1	2.3	Q2
19	*Pharmacological Reviews*	1	19.3	Q1
19	*Pharmacology Research Perspectives*	1	2.9	Q2
19	*Pharmacotherapy*	1	2.9	Q2
19	*Pharmazie*	1	1.5	Q3
19	*Therapeutics and Clinical Risk Management*	1	1.343	Q3
19	*World Journal of Clinical Cases*	1	1	Q3

**Table 3:** Factors associated with cited reference count: Results from univariate and multivariate regression analysis.

**Variable**	**Univariate *p*-value**	**Multivariable-adjusted Incidence ** **Rate Ratio**	**Multivariate *p*-value**
**Overall Categorical Effects**			
Country (Overall)	< 0.001	NA	NA
Document Type (Overall)	< 0.001	NA	NA
Years Since Publication	< 0.1	0.99	0.777
Categories (Overall)	< 0.001	NA	NA
**Country Levels**			
China (Reference)	-	-	-
USA	NA	0.88	0.408
Japan	NA	0.97	0.855
Germany	NA	0.93	0.743
South Korea	NA	1.12	0.675
UK	NA	1.60	0.083
Others*	NA	1.28	0.281
**Document Type Levels**			
Article (reference)	-	-	-
Review	NA	2.15	< 0.001
Editorial Material	NA	0.99	0.965
Letter	NA	0.23	0.001
**Category Levels**			
Anesthesiology (reference)	-	-	-
Pharmacology & Pharmacy	NA	1.53	< 0.001
Clinical Medicine & Care	NA	1.01	0.944

**Note**: *Others include Brazil, New Zealand, Netherlands, Finland, *et al*. NA: Not Applicable.

**Table 4:** Details of the top 10 most cited articles.

**Ranking**	**Title**	**First Author**	**Date and Month of Publication**	**Number of Citations**	**Journal**	**Impact Factor**	**Article ** **Type**
1	Efficacy and safety of remimazolam *versus* propofol for general anesthesia: a multicenter, single-blind, randomized, parallel-group, phase IIb/III trial a multicenter, single-blind, randomized, parallel-group, phase IIb/III trial	Doi, Matsuyuki	August 2020	292	*Journal of Anesthesia*	2.8	Article
2	A Placebo- and Midazolam-Controlled Phase I Single Ascending-Dose Study Evaluating the Safety, Pharmacokinetics, and Pharmacodynamics of Remimazolam (CNS 7056): Part I. Safety, Efficacy, and Basic Pharmacokinetics	Antonik, Laurie J.	August 2012	228	*Anesthesia and Analgesia*	4.6	Article
3	Pharmacokinetics and Pharmacodynamics of Remimazolam (CNS 7056) after Continuous Infusion in Healthy Male Volunteers: Part I. Pharmacokinetics and Clinical Pharmacodynamics	Schuettler, Juergen	April 2020	192	*Anesthesiology*	9.1	Article
4	Safety and Efficacy of Remimazolam Compared With Placebo and Midazolam for Moderate Sedation During Bronchoscopy	Pastis, NJ	January 2019	185	*Chest*	6.7	Article
5	A phase III study evaluating the efficacy and safety of remimazolam (CNS 7056) compared with placebo and midazolam in patients undergoing colonoscopy	Rex, Douglas K.	September 2018	174	*Gastrointestinal Endoscopy*	6.7	Article
6	Safety and efficacy of remimazolam in induction and maintenance of general anesthesia in high-risk surgical patients (ASA Class III): results of a multicenter, randomized, double-blind, parallel-group comparative trial	Doi, Matsuyuki	August 2020	152	*Journal of Anesthesia*	2.8	Article
7	Remimazolam tosilate in upper gastrointestinal endoscopy: A multicenter, randomized, noninferiority, phase III trial	Chen, SH	February 2021	145	*Journal of Gastroenterology and Hepatology*	3.7	Article
8	A Phase IIa, Randomized, Double-Blind Study of Remimazolam (CNS 7056) *Versus* Midazolam for Sedation in Upper Gastrointestinal Endoscopy	Borkett, Keith M.	April 2015	144	*Anesthesia and Analgesia*	4.6	Article
9	A Placebo- and Midazolam-Controlled Phase I Single Ascending-Dose Study Evaluating the Safety, Pharmacokinetics, and Pharmacodynamics of Remimazolam (CNS 7056): Part II. Population Pharmacokinetic and Pharmacodynamic Modeling and Simulation	Wiltshire, Hugh R.	August 2012	143	*Anesthesia and Analgesia*	4.6	Article
10	Remimazolam: Non-Clinical and Clinical Profile of a New Sedative/Anesthetic Agent	Kilpatrick, GJ	July 2021	138	*Frontiers in Pharmacology*	4.4	Review

**Note**: Regarding the cited data, after excluding 1,743 self-citations, the aggregate number of citations was 1,763, and the average number of citations per article was 176.3, with an average h-index of 10. The most cited article was Doi, Matsuyuki *et al.'s “Efficacy and safety of remimazolam versus propofol for general anesthesia: a multicenter, single-blind, randomized, parallel-group, phase IIb/III trial”*, published in the *Journal of Anesthesia* in May 2020, with a total citation of 292 times. In contrast, the second most cited article was about “*A Placebo- and Midazolam-Controlled Phase I Single Ascending-Dose Study Evaluating the Safety, Pharmacokinetics, and Pharmacodynamics of Remimazolam (CNS 7056): Part I. Safety, Efficacy, and Basic Pharmacokinetics*” by Antonik, Laurie J., *et al.* published in 2020 in *Anesthesia and Analgesia.*

## Data Availability

Not applicable.

## LIST OF ABBREVIATIONS

ASA: American Society of Anesthesiologists
WOS: Web of Science
IF: Impact Factor
JCR: JournaI Citation Reports
IRR: Incidence Rate Ratio
CES1: Carboxylesterase 1
GABA: Gama-aminobutyric Acidn
AChR: Nicotinic Acetylcholine Receptor
NF-κB: Nuclear Factor Kappa-Light-Chain-Enhancer of Activated B Cells
TLR4: Toll-Like Receptor 4
Nrf2: Nuclear Factor Erythroid 2–Related Factor 2
HO-1: Heme Oxygenase-1
